# Visualization and phenotyping of proinflammatory antigen-specific T cells during collagen-induced arthritis in a mouse with a fixed collagen type II-specific transgenic T-cell receptor β-chain

**DOI:** 10.1186/ar3108

**Published:** 2010-08-03

**Authors:** Patrick Merky, Tsvetelina Batsalova, Robert Bockermann, Balik Dzhambazov, Bettina Sehnert, Harald Burkhardt, Johan Bäcklund

**Affiliations:** 1 Section for Medical Inflammation Research, Department of Medical Biochemistry and Biophysics, Karolinska Institute, Scheeles väg 2, 171 77 Stockholm, Sweden; 2 Section for Medical Inflammation Research, Department of Experimental Medical Sciences, Lund University, Sölvegatan 19, 22184 Lund, Sweden; 3 Nikolaus-Fiebiger-Center for Molecular Medicine, Department of Experimental Medicine I, University Erlangen-Nürnberg, Glückstrasse 6, 91054 Erlangen, Germany; 4 Division of Rheumatology, Johann Wolfgang Goethe University Frankfurt, Theodor-Stern-Kai 7, 60590 Frankfurt am Main, Germany

## Abstract

**Introduction:**

The Vβ12-transgenic mouse was previously generated to investigate the role of antigen-specific T cells in collagen-induced arthritis (CIA), an animal model for rheumatoid arthritis. This mouse expresses a transgenic collagen type II (CII)-specific T-cell receptor (TCR) β-chain and consequently displays an increased immunity to CII and increased susceptibility to CIA. However, while the transgenic Vβ12 chain recombines with endogenous α-chains, the frequency and distribution of CII-specific T cells in the Vβ12-transgenic mouse has not been determined. The aim of the present report was to establish a system enabling identification of CII-specific T cells in the Vβ12-transgenic mouse in order to determine to what extent the transgenic expression of the CII-specific β-chain would skew the response towards the immunodominant galactosylated T-cell epitope and to use this system to monitor these cells throughout development of CIA.

**Methods:**

We have generated and thoroughly characterized a clonotypic antibody, which recognizes a TCR specific for the galactosylated CII(260-270) peptide in the Vβ12-transgenic mouse. Hereby, CII-specific T cells could be quantified and followed throughout development of CIA, and their phenotype was determined by combinatorial analysis with the early activation marker CD154 (CD40L) and production of cytokines.

**Results:**

The Vβ12-transgenic mouse expresses several related but distinct T-cell clones specific for the galactosylated CII peptide. The clonotypic antibody could specifically recognize the majority (80%) of these. Clonotypic T cells occurred at low levels in the naïve mouse, but rapidly expanded to around 4% of the CD4^+ ^T cells, whereupon the frequency declined with developing disease. Analysis of the cytokine profile revealed an early Th1-biased response in the draining lymph nodes that would shift to also include Th17 around the onset of arthritis. Data showed that Th1 and Th17 constitute a minority among the CII-specific population, however, indicating that additional subpopulations of antigen-specific T cells regulate the development of CIA.

**Conclusions:**

The established system enables the detection and detailed phenotyping of T cells specific for the galactosylated CII peptide and constitutes a powerful tool for analysis of the importance of these cells and their effector functions throughout the different phases of arthritis.

## Introduction

Collagen-induced arthritis (CIA) is the most commonly used animal model for rheumatoid arthritis. Development of CIA is dependent on both B cells and T cells. The major role of B cells is to produce collagen type II (CII)-specific antibodies, and passive transfer of such antibodies has the capacity to bind cartilage *in vivo *and induce an acute arthritis. A major role of T cells is to aid B cells in their production of anti-CII antibodies, but they are also believed to play an active part in the disease via activation of other cell types, such as synovial macrophages. The influence of T cells in established CIA, however, is less clear. Adoptive transfer of CII-specific T cells alone does not induce clinical disease but may lead to microscopic synovitis [1]. Adoptive transfer of CII-specific T cells has also been shown to prolong the otherwise acute arthritis induced by passive transfer of CII antibodies [2].

The use of T-cell receptor transgenic (TCR-tg) mice has proven a powerful tool for investigating the nature of self-reactive T cells in tolerance and autoimmunity [3]. To further facilitate the understanding for the role of T cells in CIA, three different CII-specific TCR-tg mouse strains have earlier been described and shown to display an accelerated onset of severe arthritis, compared with nontransgenic littermates. Transgenic T cells from all three strains are A^q^-restricted and recognize the same region on CII that is located between amino acid positions 260 and 270. This region harbors a lysine residue at position 264, which is naturally subjected to post-translational modifications, through hydroxylation and subsequent glycosylation. Strikingly, each of the three previously described TCR-tg mouse strains in fact recognize different forms of the CII(260-270) epitope, where the Vα11.1/Vβ8.3-tg mouse [4], the Vα11.1/Vβ8.2-tg mouse [5] and the Vβ12-tg mouse [6] respond to the nonmodified [4], the hydroxylated [7] and the galactosylated [8] CII(260-270) peptide, respectively.

Although each of the mentioned post-translationally modified peptides has its importance in A^q^-restricted CIA, we have earlier shown that glycosylation of CII is of major importance for T-cell tolerance and pathology in CIA [9]. We therefore found it important to establish an animal model that would allow for identification and tracking of T cells specific for the galactosylated CII peptide. In contrast to the Vα11.1/Vβ8.3-tg and Vα11.1/Vβ8.2-tg mouse strains, however, which express both the α-chains and β-chains of the TCR as transgenes, the galactosylated CII-specific Vβ12-tg mouse is only transgenic for the CII-specific β-chain, which may combine with any endogenous α-chain. Although the Vβ12-tg mouse displays an increased T-cell and B-cell immunity to CII and is more susceptible to CIA, it cannot be assumed that all, or even the majority, of CD4^+ ^T cells are CII specific, as was the case for the Vα11.1/Vβ8.3-tg and Vα11.1/Vβ8.2-tg mouse strains. Indeed, the Vβ12-tg mouse can also develop immunity against microorganisms as well as immunity to tuberculin-purified protein derivative, an immunogenic component of complete Freund's adjuvant (CFA) [6,8].

In the present report we have therefore established and thoroughly characterized a clonotypic antibody, which recognizes a TCR specific for the galactosylated CII(260-270) peptide in the Vβ12-tg mouse. Using this antibody we were able to describe the emerging T-cell response and its transition in Vβ12-tg mice following challenge with CII.

## Materials and methods

### Mice

To generate [B10.Q × DBA/1]F_1 _mice transgenic for Vβ12, B10.Q mice were crossed with DBA/1 mice, expressing a transgenic TCR β-chain (Vβ12), obtained from a CII-specific T-cell clone, originally derived from a CII-immunized DBA/1 mouse [6]. All animals were between 7 and 12 weeks of age at the start of the experiments. Mice were housed in a conventional animal facility and all experiments were performed according to the Swedish ethical committee guidelines.

### Antigens

The rat CII was obtained from pepsin-digested SWARM chondrosarcoma [10], and subsequently processed as previously described [11]. CII peptides, containing the 259 to 273 sequence of rat CII with a nonmodified lysine at position 264 (K264) or with a β-D-galactopyranosyl residue on L-hydroxylysine at position 264 (GalHyK264), were synthesized as previously described [12].

### Production of the recombinant 22a1-7E T-cell receptor protein

Total RNA of the murine T-cell hybridoma clone 22a1-7E was extracted and cDNA synthesis was performed following standard protocols. The V-gene segments encoding the Vα16 and Vβ12 chains of the 22a1-7E TCR were amplified by PCR using sequence-specific primers. Following an earlier-described method for generation of single-chain Fv antibodies [13], we used an assembly PCR strategy to connect the Vα16 segment and the Vβ12 segment by a fragment encoding a glycine-serine-linker (-(GSSS)_4_-) and cloned the entire construct into the pTriEx-1.1 vector (Merck, Darmstadt, Germany).

Upon control by nucleotide sequencing, the respective construct was transformed into Origami™ (DE3)pLacI cells (Merck, Darmstadt, Germany) for bacterial expression. The 8 × His-tagged recombinant TCR was purified from inclusion bodies of the lyzed bacteria by affinity chromatography on a Co^2+^-affinity resin (Takara Bio Europe/Clontech, Saint-Germain-en-Laye, France). The purification was performed according to the manufacturer's instructions and the protein was eluted with 150 mM imidazole buffer containing also 6 M guanidinium hydrochloride, 50 mM sodium phosphate, 500 mM NaCl, 3 mM β-mercaptoethanol, pH 7.8. Purified fractions of the denatured TCR protein were pooled and diluted to 300 μg/ml for the subsequent refolding procedure, consisting of repetitive dialysis steps. A mixture of 0.5 mM GSSH and 5 mM GSH was added and stirred for 1 hour at 4°C. The preparation was subsequently dialyzed for two sequential 16-hour intervals in 100 mM Tris, 400 mM arginine, 2 mM β-mercaptoethanol, 2 mM EDTA, pH 8.0 and 100 mM Tris, 200 mM arginine, 2 mM β-mercaptoethanol, 2 mM EDTA, pH 8.0. Subsequently, the buffer was changed to 20 mM Tris, 150 mM NaCl, 1 mM β-mercaptoethanol, 2 mM EDTA, pH 8.0 and the dialysis procedure continued for another 32 hours (2 × 16 hours).

Homogeneity of the respective preparations was demonstrated by the detection of a single protein band with an electrophoretic mobility corresponding to calculated molecular mass in 14% acrylamide gels upon Coomassie Blue staining.

### Generation of a monoclonal antibody specific for the 22a1-7E T-cell receptor

To generate clonotypic antibodies, E3 rats were immunized with a recombinant 22a1-7E TCR protein in CFA (Difco, Detroit, MI, USA). The 22a1-7E TCR originates from a T-cell clone, specific for the galactosylated CII(260-270) epitope, isolated from a CII-immunized Vβ12-tg mouse. Lymph node cells from E3 rats immunized with the 22a1-7E TCR protein were prepared 10 days after immunization and were fused with myeloma cells (P3X63-Ag8.653) as described previously [14]. Supernatant from growing cultures was tested for staining of the 22a1-7E T cell hybridoma clone (Vα16/Vβ12; RB, unpublished data), HCQ.3 (Vα16/Vβ8 [15]) and HCQ.4 (Vα4/Vβ12 [15]). Cells producing antibodies that stained 22a1-7E, but not HCQ.3 or HCQ.4 hybridomas, were selected for expansion, subcloning and retesting. After two additional rounds of selections, one clone specific for the 22a1-7E TCR of the IgG_1 _isotype was obtained and was denoted B22a1.

### Antibodies and flow cytometry analysis

The TCR binding specificity of B22a1 and the expression level of TCR on the surface of T-cell hybridoma was determined by flow cytometry using the following antibodies and reagents: B22a1-bio (produced from our in-house collection), TCR cβ-bio (clone H57-597; in-house collection) and propidium iodide (Invitrogen, Eugene, Oregon, USA). Subsequently biotin was detected by allophycocyanin-conjugated streptavidin (BD Pharmingen, San Diego, CA, USA). The cells were then acquired with a flow cytometer (FACSort; BD Biosciences, San Jose, CA, USA) by gating on propidium-iodide-negative live cells.

For determination of relative frequencies of B22a1^+ ^T cells, their activation and cytokine profile, the following antibodies were used for extracellular staining: Anti-Fcγ receptor (2.4G2) and B22a1-bio from our in-house collection; anti-CD11b-FITC (M1/70), anti-CD45R-FITC (RA3-6B2), anti-MHC class II-FITC (7.16.17), anti-CD49b-FITC (DX5), and antigen-presenting cell (APC)-conjugated streptavidin from BD Pharmingen (San Diego, CA, USA); and anti-CD4-PE-Cy5.5 (RM4-5) from eBioscience (San Diego, CA, USA). For intracellular staining, the following antibodies were used: anti-CD40L-PE (MRI), anti-IFNγ-PE-Cy7 (GMX1.2) and anti-IL-17-Pacific blue (TC11-18H10.9) purchased from eBioscience (San Diego, CA, USA), and anti-IL2-Alexa Fluor 700 (JES6-5H4) purchased from BioLegend (San Diego, CA, USA).

All antibodies were titrated for optimal saturating concentration. For exclusion of dead cells, the LIVE/DEAD Fixable Green Dead Cell Stain Kit (Invitrogen, Eugene, Oregon, USA) was used. At least 1 × 10^5 ^Th cells were acquired on a flow cytometer (FACS Aria/FACS LSRII; BD Biosciences, San Jose, CA, USA) and were analyzed using FlowJo software (Treestar, Inc. Ashland, OR, USA).

For antigen-specific detection of CD154 (CD40L) and cytokines, single-cell suspensions were processed as described earlier by Frentsch and colleagues [16] with some modifications. In brief, 1.2 × 10^7 ^cells/ml were cultured for 6 hours in the presence of the galactosylated CII peptide (GalHyK264, 10 μg/ml) and anti-CD28 (1 μg/ml, clone 61109; R&D Systems, Minneapolis, MN, USA). For intracellular accumulation of CD40L and cytokines, 2 μg/ml Brefeldin A (Sigma-Aldrich, St. Louis, MO, USA) was added after 2 hours of culture. The cells were then washed and stained for 15 minutes at room temperature with the surface markers. Afterwards, the cells were fixed with Cytofix/Cytoperm solution (BD Pharmingen, San Diego, CA, USA) and permeabilized with Perm/Wash buffer (BD Pharmingen, San Diego, CA, USA) according to the manufacturer's instructions, and were stained for CD40L and cytokines.

### Monoclonal antibody treatments and lymphocyte assay

The antibody B22a1 was purified from culture supernatant using gamma-bind plus sepharose gel matrix (GE Healthcare, Uppsala, Sweden), dialyzed against PBS, sterile filtered, and kept at -80°C. Antibody concentration was determined either spectrophotometrically (at 280 nm) or by freeze-drying.

For *in vitro *depletion, single-cell suspensions from pooled popliteal and inguinal lymph nodes were prepared 10 days post immunization. One-half of each single-cell suspension was depleted of B22a1^+ ^T cells, whereas the remaining half was kept untouched on ice for further processing. Briefly, lymphocytes were incubated with biotinylated antibody B22a1 for 20 minutes at 4°C. Subsequently, biotin binder Dynabeads (Invitrogen Dynal AS) were added according to the manufacturer's recommendation and labeled cells were depleted by placing the sample tubes in a magnetic stand. The samples were depleted twice to reach a final purity > 95% before both the untouched and depleted fractions were set up into triplicate cultures. The cells were stimulated with antigen (lathyritic CII (pepsin-free), K264, GalHyK264 and ConA) at a concentration of 10^6 ^cells/well in microtiter plates and were incubated for 72 hours. One microcurie of methyl-[^3^H]thymidine (Amersham, GE Healthcare, Little Chalfont, Buckinghamshire, UK) was added to each well for an additional 15 to 18 hours. Cell proliferation was measured by counting the incorporation of methyl-[^3^H]thymidine.

For *in vivo *depletion, B22a1 was injected intraperitoneally as a single dose of 240 μg/mouse. To increase the depletion capacity, MAR18.5 was administered as a secondary antibody 1 hour after the primary antibody by the same method in a dose of 340 μg [17]. The monoclonal antibodies were given at day 0 (4 hours before immunization), day 6 or day 20, depending on the experiment.

### Induction and evaluation of CIA and anti-CII antibodies

Mice were injected by the base of the tail with 50 μl emulsion consisting of 100 μg rat CII emulsified 1:1 in CFA (Difco, Detroit, MI, USA). Development of clinical arthritis was followed three times weekly through visual scoring of the paws, starting 2 weeks after immunization. The arthritis was scored using a scale ranging from 1 to 15 for each paw, with a maximum score of 60 per mouse [18]. Each arthritic toe and knuckle was scored as 1, with a maximum of 10 per paw. A score of 5 was given to an arthritic ankle.

Blood samples were collected on days 17 and 35 for determination of anti-CII antibody responses. The titers of total anti-CII IgG as well as the IgG_1_, IgG_2a_, IgG_2b _and IgM isotypes were determined through quantitative ELISA [19], where serum was titrated (1:10 to 1:10^6^) in parallel to the standard and titer values were interpolated within the linear range and related to the standard curve. Biotinylated rat anti-mouse IgGκ (clone 187.1; our collection) or peroxidase-conjugated goat anti-mouse antibodies specific for IgM, IgG_1 _or IgG_2a _(Southern Biotech, Birmingham, AL, USA) were used as detecting antibodies. Binding of biotinylated antibodies was revealed by Extravidin Peroxidase (Sigma-Aldrich). Plates were developed using ABTS (Roche Diagnostics, Mannheim, Germany) as substrate, and the absorbance was measured at 405 nm (Synergy-2; BioTek, Winooski, VT, USA). Total anti-CII IgG levels were measured (μg/ml) using purified polyclonal anti-CII IgG antibodies of a known concentration as a standard. Isotype levels were measured as arbitrary concentrations using our purified monoclonal anti-CII antibodies with known isotype or pooled sera from arthritic mice.

### T-cell hybridoma assays

All cells were cultured in DMEM + Glutamax-I (Gibco Life Technologies, Grand Island, NY, USA) supplemented with 5% heat-inactivated FCS and penicillin/streptomycin.

For antibody-induced T-cell hybridoma stimulation, flat-bottomed microtiter 96-well plates (NUNC, Thermo Fisher Scientific, Roskilde, Denmark) were precoated with a titrated concentration of purified immunoglobulins (B22a1, anti-TCR cβ, anti-CD4 (clone H129.19), all produced from in-house hybridomas) starting at 5 μg/ml. Plates were then washed and 5 × 10^4^/well T-cell hybridoma were added and cultured for 24 hours, before assaying the supernatant for IL-2 content.

For the inhibition of antigen-induced T-cell hyridoma stimulation, 5 × 10^4^/well T-cell hybridoma where pre-incubated with a titrated concentration of purified immunoglobulins (B22a1, anti-TCR cB (clone H57-597), anti-CD4 (clone H129.19; our collection)) starting at 10 μg/ml in flat-bottomed microtiter wells in 50 μl medium. After 1 hour of incubation at 37°C, the hybridoma were washed twice with PBS and transferred to a previously prepared microtiter plate containing splenocytes as APCs (5 × 10^5^/well) and peptide (0.3 μg/ml). After 24 hours of incubation at 37°C, the supernatants were analyzed for IL-2.

To determine the stimulatory capacity of the clonotypic antibody, the relative production of IL-2 between nonstimulated and stimulated T-cell hybridoma clones was determined by ELISA. Hereby, 50 μl supernatant were removed from the plates after 24 hours of culture. For detection, Jes6-1A12 (5 μg/ml; our collection) and Jes6-5H4-biotin (2 μg/ml; Mabtech, Nacka Strand, Sweden) were used as the capture antibody and detection antibody, respectively. Binding of biotinylated antibodies was revealed by Europium-labeled streptavidin (PerkinElmer Life Sciences, Inc. Boston, MA, USA) and plates were analyzed using a Victor 1420 multi-label counter (PerkinElmer Life Sciences, Inc. Boston, MA, USA).

### Statistical analysis

Antibody levels and *in vitro *lymphocyte assays were analyzed with the Mann-Whitney U test, whereas arthritis severity was analyzed with Fisher's exact test and the flow cytometry data with an unpaired *t *test.

## Results

### Characterization of B22a1 specificity

The clonotypic antibody B22a1 was generated through immunization of a rat with a recombinant protein, corresponding to the TCR of the 22a1-7E T-cell clone, originating from a CII-primed DBA/1-Vβ12-tg mouse. The purified B22a1 antibody was tested for binding to the 22a1-7E T-cell hybridoma clone as well as to other A^q^-restricted and CII-specific clones sharing either one or none of the TCR chains with the 22a1-7E clone (Vα16/Vβ12). In addition, two more closely related T-cell hybridoma clones (18b4-10 D and 3D4-1.2) were also tested. The two latter clones were also obtained from the DBA/1-Vβ12-tg mouse and share both TCR α-chains and β-chains with 22a1-7E, but differ at one single amino acid within the complementarity determining region 3 (CDR3) region of the nontransgenic Vα chain (RB, unpublished data).

**Figure 1:**
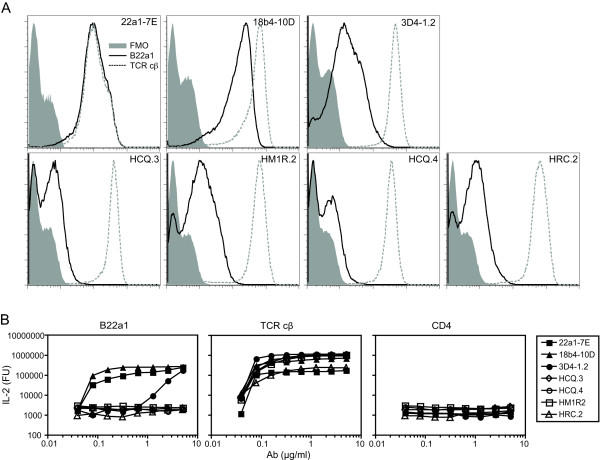
**B22a1 binding requires a combination of Vβ12 and Vα16 T-cell receptor chain**. **(a) **Flow cytometric analysis of T-cell hybridoma expressing different Vα-chain and Vβ-chain combinations stained with the biotinylated antibody B22a1 and allophycocyanin-conjugated streptavidin. Histograms show the staining intensities of negative control (FMO; stained with allophycocyanin-conjugated streptavidin only), B22a1 (straight line) for specificity and T-cell receptor (TCR) cβ (dotted line) for TCR surface expression levels. **(b) **For stimulation, T-cell hybridomas were cultured for 24 hours in precoated cell culture plates with titrated amounts of B22a1, TCR cβ (positive control) and CD4 (negative control), whereupon IL-2 production was determined by ELISA. Ab, antibody; FU, fluorescence units.

The B22a1 antibody failed to stain the T-cell clones not expressing a Vα16 chain together with the transgenic DBA/1-Vβ12-tg chain (HCQ.3, HCQ.4, HRC.2 and HM1R.2; Figure 1a). From the clones expressing the DBA/1-Vβ12-tg chain together with a closely related Vα16 chain, the B22a1 antibody stained the 22a1-7E and 18b4-10 D T-cell clones strongly but not the 3D4-1.2. Also, when normalizing the B22a1 staining against the level of surface TCR expression, significant staining was only observed against the 22a1-7E and 18b4-10 D clones with a sevenfold stronger staining against the 22a1-7E T-cell hybridoma clone (Table 1). Comparison of the amino acid sequence within the CDR3 region of the Vα16 chain shows Met-106 (22a1-7E) and Tyr-108 (22a1-7E and 18b4-10D) to be involved in B22a1 recognition, as substitution of these to either Ile-106 (18b4-D10) or Asp-108 (3D4-1.2) leads to decreased and insignificant antibody binding, respectively.

**Table 1:** Binding specificities of the B22a1 antibody towards different T-cell receptors

Clone	TCR Vα chain	TCR Vβ chain	Antigen specificity	SI	TCR cβ geometric mean	**SI normalized to TCR**^**a**^
22a1-7E	16	12	GalHyK264	24.0	110	218.1
18b4-10D	16	12	GalHyK264	12.3	431	28.6
3D4-1.2	16	12	GalHyK264 and GlcGalHyK264	1.1	431	2.5
HCQ.3	16	8.1	GalHyK264	0.1	357	0.2
HM1R.2	4	12	GalHyK264	0.9	505	1.7
HCQ.4	4	12	K264	0.1	316	0.3
HRC.2	2	20	K264	0.3	591	0.5

Table showing the staining index (SI) of B22a1 and the SI normalized to the T-cell receptor (TCR) expression, respectively. GalHyK264, CII(260-270) with a β-D-galactopyranosyl residue on L-hydroxylysine at position 264; GlcGalHyK264, CII(260-270) with an α-D-glucopyranosyl-(1 > 2)-β-D-galactopyranosyl residue on L-hydroxylysine at position 264; K264, CII(260-270) with a nonmodified lysine at position 264; TCR cβ, anti-TCR cβ antibody (clone H57-597). ^a ^SI normalized to TCR is multiplied by 1,000.

To test the stimulatory capability of the B22a1 antibody, T-cell hybridomas were incubated with precoated B22a1 antibody and monitored for activation through IL-2 production (Figure 1b). In agreement with the staining data, B22a1 were highly effective in stimulating the 22a1-7E and 18b4-10 D clones. B22a1 could also stimulate the 3D4-1.2 clone at high concentration but failed to activate CII-specific clones not expressing the Vα16/Vβ12 TCR combination. In an inhibition assay, the B22a1 antibody could also inhibit activation of the 22a1-7E and 18b4-10 D clones as well as partially block activation of the 3D4-1.2 clone, but not that of unrelated clones (data not shown), again showing that the B22a1 antibody is highly specific for the Vα16/DBA/1-Vβ12-tg TCR combination. Finally, the B22a1 antibody failed to stain A^q^-restricted T cells from mice immunized with control (non-CII) antigens as well as an A^q^-restricted T-cell hybridoma clone specific for pepsin (see Additional file 1).

### B22a1-binding T cells dominate CII-specific response in Vβ12-tg mice

**Figure 2:**
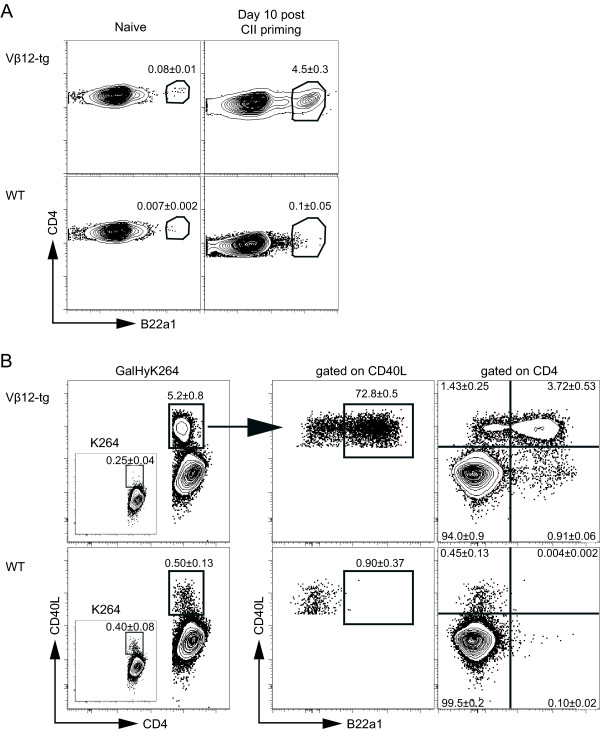
**B22a1^+ ^T cells dominate the collagen type II-specific T-cell expansion in Vβ12 transgenic mice**. Lymph nodes of naïve and collagen type II (CII)-primed mice 10 days post immunization were restimulated for 6 hours in the presence of GalHyK264 and Brefeldin A. Flow cytometry was subsequently performed to determine B22a1 frequencies and CD40L upregulation. **(a) **Dot plots show B22a1 mean frequency among CD4 cells in Vβ12-transgenic (Vβ12-tg) mice (*n *= 5) and wildtype (WT) littermates (*n *= 4 and *n *= 5) left untreated (naïve) or immunized 10 days earlier. **(b) **Left panel: percentage of CD4 T cells upregulating CD40L upon restimluation with GalHyK264 in immunized Vβ12-transgenic mice and WT littermates. Insert: frequency of CD40L upregulation following stimulation with the nonmodified K264-peptide. Middle panel: distribution of B22a1 staining among CD4^+^CD40L^+ ^T cells. Right panel: quadrant gates of CD4-gated T cells. Numbers indicate frequencies (mean ± standard error of the mean) of two individual mice per group, reproduced in at least two independent experiments (b).

Having shown that the B22a1 antibody recognizes CII-specific T-cell clones obtained from CII-immunized Vβ12-tg mice, we next set out to determine the relative frequency of T cells expressing the clonotypic determinant among CII-reactive T cells in primed and nonprimed Vβ12-tg mice. In nonimmunized Vβ12-tg mice, the frequency of B22a1^+^CD4^+ ^T cells in peripheral lymph nodes and spleen was 0.08%, compared with around 0.007% in wildtype littermates (Figure 2a). Ten days after immunization, the frequency in primed Vβ12-tg mice had increased to 4.5% of CD4^+ ^T cells in draining lymph nodes, while remaining at a low frequency of 0.1% in littermate controls.

To determine the relative frequency of B22a1^+^CD4^+ ^T cells among the CII-reactive CD4^+ ^T cells we used the expression of CD154 (CD40L) as a generic marker of antigen-specific T cells [16]. Around 5 to 6% of CD4^+ ^T cells expressed CD40L upon restimulation *in vitro *with the galactosylated CII peptide, and 70 to 80% of these were also positive for the B22a1 antibody (Figure 2b, middle panel). Roughly 20% of the B22a1^+ ^T cells did not express CD40L, however, and hence appeared to remain in a nonactivated state (Figure 2b, right panel). As expected, mice devoid of the transgenic CII-specific Vβ12 chain mounted a much weaker immune response against the galactosylated CII peptide upon restimulation *in vitro*, and only very few B22a1^+^CD40L^+^CD4^+ ^T cells were identified. As reported earlier [8], the recall response of Vβ12-tg mice was strongly biased to the galactosylated CII peptide with only a minimal response directed against the nonglycosylated CII peptide (denoted K264; Figure 2b, left panel insert). Nontransgenic littermates, on the other hand, mounted a low but significant response to both peptides.

**Figure 3:**
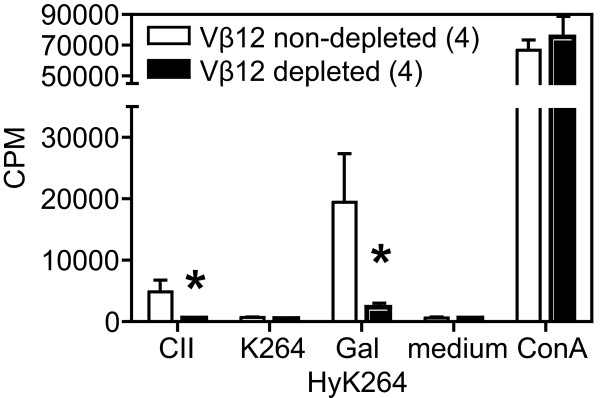
***In vitro *depletion of B22a1^+ ^T cells reduces recall response to collagen type II**. Vβ12-transgenic mice were immunized with collagen type II (CII) and 10 days later lymph node cells were depleted of B22a1^+ ^T cells *in vitro*, and alterations in the CII-specific [H^3^]thymidine incorporation were noted after 4 days of culture with lathyritic CII, K264 peptide, GalHyK264 peptide, medium alone and ConA. Data show mean ± standard error of the mean of four individual mice before and after depletion. *P < 0.05. CPM, counts per minute.

To further analyze the significance of B22a1^+ ^T cells in Vβ12-tg mice, lymph node cells from CII-primed Vβ12-tg mice were depleted of B22a1^+ ^T cells *in vitro *and alteration in the CII-specific recall response was subsequently determined (Figure 3). *In vitro *depletion of B22a1^+ ^T cells resulted in a reduced recall response to CII and the galactosylated CII peptide by approximately 80%, hence confirming the *ex vivo *data showing B22a1^+ ^T cells to predominate the CII-specific T-cell response in Vβ12-tg mice.

### CII-specific T cells expand in the priming phase and retract as the first arthritis symptoms appear

Having shown that the B22a1^+ ^T-cell population predominates the CII-specific T-cell population in Vβ12-tg mice, we next analyzed the dynamics of antigen-specific T cells during CIA by determining the frequency of the B22a1^+ ^T cells at different time points after induction of CIA. To relate the frequency of antigen-specific T cells to development of arthritis, a cohort of Vβ12-tg mice was immunized with CII and followed for clinical disease in parallel to the *ex vivo *analyses of representative animals.

**Figure 4:**
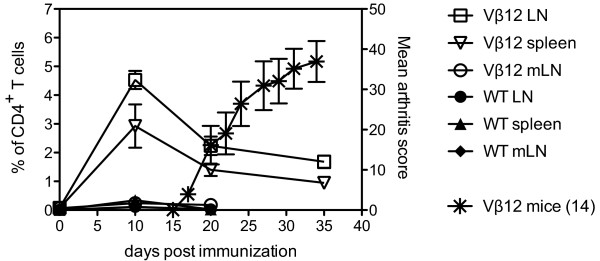
**Collagen type II-specific T cells expand in priming phase and retract when arthritis symptoms appear**. The indicated numbers of Vβ12-transgenic mice were immunized with collagen type II (CII) and were followed for development of arthritis (stars, referring to right *y *axis). In parallel, frequencies of B22a1^+ ^T cells in CII-primed Vβ12-transgenic mice and wildtype (WT) littermates were assessed by flow cytometry in draining lymph nodes (LNs, open squares and closed circles), spleen (open and closed triangles) and mesenteric lymph node (mLN, open circles and closed diamonds) (referring to left *y *axis). Data show the mean ± standard error of the mean of four individual mice (WT littermates), five individual mice (Vβ12-transgenic) and 14 individual mice (day 35).

Immunization of Vβ12-tg mice with heterologous CII emulsified in CFA induced an onset of arthritis around day 17 that rapidly progressed to severe disease within the following 2 weeks (Figure 4, right *y *axis stars). *Ex vivo *analyses clearly showed that CII priming at the base of the tail triggered a primary immune response in the draining inguinal lymph nodes, leading to an increased frequency from initially 0.08% of B22a1^+^CD4^+ ^T cells in nonimmunized animals up to 4.5% within the first 10 days (Figure 4, left *y *axis). Twenty days post immunization, however, the B22a1^+^CD4^+ ^T-cell frequency subsequently dropped again to 2.2% and slowly stabilized around 1.7% at day 35 (Figure 4, left *y *axis). A similar pattern could be observed in the spleen, albeit with a somewhat lower frequency. Measurement of the mesenteric lymph node, however, showed that only 0.2% of B22a1^+^CD4^+ ^T cells infiltrated this organ at peak day 10-indicating that the homing B22a1^+^CD4^+ ^T cells did not occur at random and that the mesenteric lymph node is not involved in the arthritis-specific immune mechanisms, since these cells had retracted to approximate base levels again at day 20. It was also possible to detect significantly increased frequencies of B22a1^+ ^T cells in wildtype littermates 10 days after immunization (Figure 4). Frequencies were profoundly reduced (0.1 and 0.3% in lymph nodes and spleen, respectively), however, compared with those observed in Vβ12-tg mice.

### The anti-CII response is dominated by proinflammatory cytokine producing B22a1^+ ^T cells during arthritis

To extend our analysis on the dynamics of CII-specific T cells in Vβ12-tg mice during development of CIA, we next investigated the activation status and production of proinflammatory cytokines of CII-specific T cells at different time points after immunization with CII. We also wanted to include B22a1^-^CD4^+ ^T cells in these analyses to ensure that our *ex vivo *data on the frequency of B22a1^+^CD4^+ ^T cells could be extrapolated to the whole CII-specific T-cell repertoire in Vβ12-tg mice. To achieve this, CII-primed Vβ12-tg mice were sacrificed at three different time points during CIA. Pooled draining lymph nodes and the spleen were then analyzed for CD40L and cytokine expression in the priming phase (day 10), shortly after arthritis onset (day 25) and, finally, during severe clinical arthritis (day 35).

**Figure 5:**
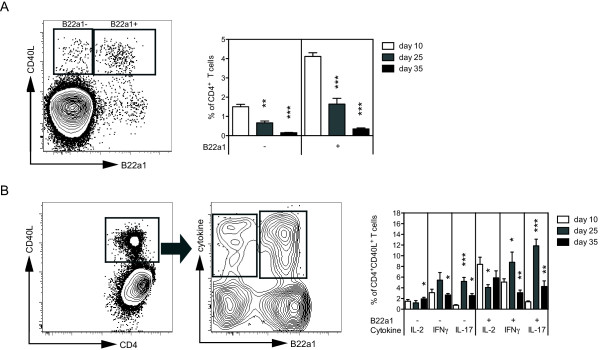
**B22a1^+ ^T cells dominate during arthritis and are the main producers of proinflammatory cytokines**. Vβ12-transgenic mice were primed with collagen type II (CII) and lymphocytes were analyzed 10, 25 and 35 days after immunization, following 6 hours of restimulation in the presence of the GalHyK264 peptide and Brefeldin A. **(a) **Representative dot plot from day 35 analysis and a summarizing bar graph showing the frequencies of CD4^+ ^T cells being B22a1^-^and B22a1^+ ^and expressing CD40L. **(b) **Cytokine production of activated CII-specific T cells, determined by gating on total CD40L^+^CD4^+ ^T cells. Bars represent mean percentage (± standard error of the mean) of cytokine-producing cells among CD4^+^CD40L^+ ^T cells, stratified according to B22a1 expression. IL-2 bars show frequency of cells producing IL-2 only, whereas IFNγ and IL-17 bars include IFNγ and IL17 single positives as well as IFNγ/IL-2 and IL-17/IL-2 double positives, respectively. The IFNγ and IL-17 bars are not adjusted for IFNγ/IL-17 double-positive cells due to their low abundance. All plots include 12 mice, eight mice and five mice for day 10, day 25 and day 35, respectively. *P < 0.05, **P < 0.01, ***P < 0.001.

The CD40L expression in CD4^+ ^T cells followed the same pattern as observed for the frequency of B22a1^+^CD4^+ ^T cells, with the most frequent expression detected at day 10 and with fading frequencies at later time points. Importantly, the B22a1^+^CD4^+ ^T cells prevailed as the predominant CII-specific T-cell population, constituting 75 to 80% of all CD40L^+^CD4^+ ^T cells, throughout the disease course (Figure 5a).

Having shown that the frequency of activated CII-specific T cells in the draining lymph nodes decline from day 10 and onwards (Figures 4 and 5a), we were also interested in evaluating the quality of the CII-specific T-cell response over time with regard to production of proinflammatory cytokines, as these have previously been found to dominate the response in Vβ12-tg upon immunization with CII in CFA [8,20]. We therefore stratified the B22a1^+ ^and B22a1^- ^T-cell responses according to their production of IL-2, IFNγ and IL-17 to detect whether the relative abundance of these cells would shift over time among the CII-specific CD40L-expressing T cells (Figure 5b). Indeed, stratification of CII-specific T cells showed that both B22a1^+ ^and B22a1^- ^T cells mounted an IFNγ and IL-2 response that was maintained in the draining lymph node from day 10 post immunization and throughout the development of clinical arthritis. Compensating for the reduction in the total frequency of CII-specific T cells from day 10 onwards (Figure 5a), however, showed that the frequency of IL-2-producing and IFNγ-producing T cells among CD4^+ ^T cells was highest at the priming phase (day 10) and then decreased with the progressing arthritis in both B22a1^+ ^and B22a1^- ^CD4^+ ^T cells (data not shown). In marked contrast, CII-specific IL-17 producing T cells occurred with low frequency in the priming phase and instead peaked at the time of disease onset whereupon the frequency dropped again to low levels by the time of severe disease (Figure 5b). This delay in occurrence was further emphasized by comparing the frequency of IL-17-producing T cells among CD4^+ ^T cells (data not shown).

Importantly, cytokine production was only detected in cells that had upregulated CD40L. In addition, on average 0.4%, 1.6% and 0.6% of the CD40L-expressing T cells were found to produce both IL-17 and IFNγ during the priming, disease-onset and later phases, respectively (data not shown). Determination of the frequency of IL-2-producing, IL-17-producing and IFNγ-producing T cells, however, could only account for 20 to 35% of the total CII-specific T-cell population. These data collectively show that, following priming *in vivo*, the CII-specific T-cell response in the draining lymph nodes is initially dominated by Th1. As the overall T-cell response subsequently fades, however, the T-cell response shifts to also include Th17 by the time of onset of clinical disease. Still, 60 to 85% of the antigen-specific response could not be accounted for by measuring the production of IL-2, IL-17 and IFNγ. This observation suggests that additional effector and regulatory functions are probably operating through the remaining CII-specific T cells in order to control the progression and regulation of disease.

**Figure 6:**
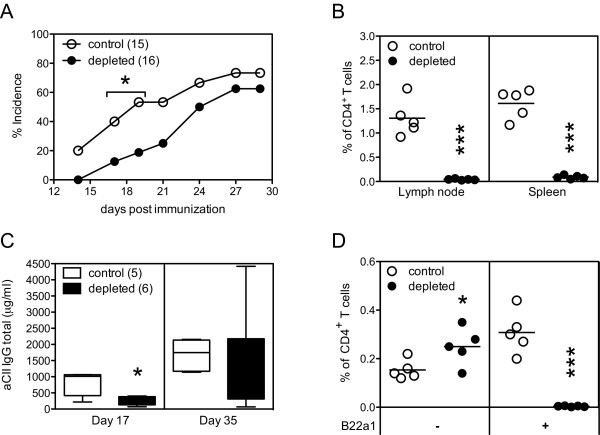
**Efficient depletion of B22a1^+ ^T cells before arthritis onset has limited effect on clinical disease**. **(a) **The indicated numbers of Vβ12-transgenic male mice were treated with B22a1 and MAR18.5 antibodies 4 hours prior to immunization with collagen type II (CII) and followed for development of arthritis. **(b) **Frequencies of B22a1^+ ^T cells by the end of the arthritis experiment were assessed by flow cytometry in the lymph nodes and spleen. **(c) **Anti-CII IgG antibody levels at days 17 and 35 after immunization are shown as box plots. **(d) **Scatter plot showing the frequencies of CD4^+ ^T cells being B22a1^- ^and B22a1^+ ^and expressing CD40L after 6 hours of restimulation in the presence of the GalHyK264 peptide and Brefeldin A by the end of the arthritis experiment at day 35. *P < 0.05, **P < 0.01, ***P < 0.001.

Data thus far suggest that both B22a1^+ ^and B22a1^- ^CD4^+ ^T cells could be involved in the development of CIA in Vβ12-tg mice. To confirm this, we depleted B22a1^+^CD4^+ ^T cells *in vivo *prior to immunization with CII and subsequently observed the mice for alternation in the development of CIA. Indeed, *in vivo *treatment with the B22a1 antibody was efficient in depleting B22a1^+^CD4^+ ^T cells, as only very few B22a1^+ ^T cells could be identified in the spleen and lymph nodes by the end of the arthritis experiment (Figure 6b) and caused a delay in disease onset (*P *< 0.05; Figure 6a). In agreement with clinical disease, analysis of the anti-CII antibody titers from the experiments also showed an initial delay of antibody production that was later recovered as arthritis had developed (Figure 6c).

We also investigated IgM, IgG_1_, IgG_2a _and IgG_2b _anti-CII antibodies following antibody treatment, to see whether depletion of B22a1^+^CD4^+ ^T cells would result in a skewing in the quality of the antibody response. A significant reduced IgM response was observed in mice depleted of B22a1^+^CD4^+ ^T cells on day 17 (*P *= 0.009) but not on day 35 (*P *= 0.75; data not shown). Even though levels of anti-CII IgG_1_, IgG_2a _and IgG_2b _levels were uniformly reduced in depleted mice at day 17, the differences did not reach statistical significance (*P *= 0.178, *P *= 0.082 and *P *= 0.125 for IgG_1_, IgG_2a _and IgG_2b_, respectively; data not shown). Analysis of the frequency of CII-specific T cells by the end of the arthritis experiments showed that the B22a1^-^CD4^+ ^T cells in depleted mice had expanded compared with nontreated control mice (*P *= 0.038). Together with the data on antibody isotype levels and the stratified cytokine profiles, this suggests that B22a1^- ^and B22a1^+ ^T cells exhibit similar qualitatively effector functions and that B22a1^- ^T cells indeed could take over the role of the depleted B22a1^+ ^T cells in Vβ12-tg mice (Figure 6d).

## Discussion

The galactosylated CII(260-270) peptide has been shown to constitute the immunodominant T-cell epitope in A^q^-expressing mice [12,15], and T cells specific for the galactosylated variant of the CII(260-270) epitope have been shown to be of major importance for development of arthritis [9]. In the present report, we have used the Vβ12-tg mouse to establish a system where it is possible to track T cells specific for galactosylated CII peptide. The Vβ12-tg mouse is so far the only described TCR-tg mouse with increased cellular immunity to galactosylated CII and increased susceptibility to CIA [6,8]. Despite the transgenic Vβ12 chain being expressed on virtually all CD3^+ ^T cells, however, it may still recombine freely with endogenously derived α-chains, and the frequency of CII-specific T cells in the Vβ12-tg mouse was therefore not known.

Recombinant MHC class II tetramers/multimers presenting CII peptides have previously been used to identify CII-specific T cells *ex vivo *in A^q^-expressing [21,22], DR4-expressing [23], and DR1-expressing mice [24]. Successful identification of CII-specific T cells in A^q^-expressing mice, however, required that the CII peptide had been covalently linked to the recombinant MHC class II molecule. Covalent linkage of the peptide is used in order to increase the stability of the CII peptide:MHC class II molecule complex [25]. This is presently not a realistic option for the galactosylated CII peptide as hydroxylation of lysine residues of recombinant CII is absent or poor in prokaryotic, yeast and insect cell systems [26,27] but is necessary for subsequent galactosylation of hydroxylysine residues. Because of the poor feasibility to detect galactosylated CII-specific T cells using the MHC class II tetramer technology, we instead decided to generate a clonotypic antibody recognizing T cells specific for the galactosylated CII peptide in the Vβ12-tg mouse. Hereby we could show that B22a1^+ ^T cells constitute only a minority of the total TCR repertoire. Still, by performing depletion experiments *in vitro *and through combinatory analysis of the frequencies of clonotypic T cells and the activation status of the total CII-specific T-cell population *ex vivo*, we found that the B22a1 antibody recognizes the vast majority of the CII-specific T cells in Vβ12-tg mice.

The frequency of B22a1^+ ^T cells in naïve Vβ12-tg mice (~0.1%) was found to be considerably lower than that reported for other TCRβ-tg mice using MHC class II tetramer/multimers (1 to 3% [28-30], but still around 10 times higher than in wildtype littermates). This is a considerably higher frequency than that estimated for a specific T-cell clone in a naïve wildtype mouse [31], but may partly be explained by the fact the B22a1 antibody could recognize T cells with closely related but distinct TCRs.

Following priming of Vβ12-tg mice with CII *in vivo*, we observed a 50-fold increase in the frequency of B22a1^+ ^T cells - and the majority (80%) of, but not all, B22a1^+ ^T cells would acquire an activated phenotype. While our data consistently indicated CII-specific B22a1^+ ^and B22a1^- ^T cells to exhibit almost identical phenotypes and only differ in their frequency of occurrence, it may be assumed that a similar proportion of nonactivated T cells can be found within the CII-specific B22a1^- ^fraction. The lack of activation in 20% of the cells is probably explained by the nonphysiological frequency of CII-specific T cells, leading to competition for APCs, as has been demonstrated in other studies [32-34]. Owing to the limited number of APCs and the accessibility of MHCs and costimulatory molecules *in vitro*, a more stringent selection for activation may be imposed despite an excess of the galactosylated CII peptide.

Our results support an important role for CII-specific T cells in the early phase of the arthritogenic immune response (reviewed in [35]), where depletion of the predominant B22a1^+ ^T-cell population caused a delayed onset of clinical arthritis. CII-specific T cells not targeted by the clonotypic antibody were found to expand rapidly and to partially replace the depleted B22a1^+ ^T-cell subset, however, which was also evident when analyzed at the end of the arthritis experiment. It is of note that depletion with the B22a1 antibody before priming with CII caused a strong and rapid as well as longlasting reduction in the frequency of B22a1^+ ^T cells. We found no evidence that antibody treatment would cause TCR downregulation, as has been shown for γδ-T cells after treatment with a monoclonal antibody specific for the constant region of the γδ-TCR and which caused downregulation of the TCR and generation of persistent invisible clones with reduced responsiveness [36] (data not shown). Moreover, the monoclonal antibody MAR18.5 was administered as a secondary antibody after B22a1 to increase the depletion capacity as previously shown [17]. Finally, sham-operated and thymectomized mice subjected to antibody treatment and subsequent immunization appeared equally protected from CIA, compared with nondepleted mice, excluding a role for recent B22a1^+^CD4^+ ^thymic emigrants in the later-occurring arthritis (data not shown). Taking these observations together, therefore, CII-specific B22a1^- ^T cells that are not depleted by the B22a1 antibody seem to be sufficient to trigger CIA.

Although not conclusively demonstrated, an active role for CII-specific T cells during clinical arthritis is indirectly supported by their presence in the arthritic joints [23,24], and also by experimental therapies specifically targeting Th1 and Th17 during active CIA [35,37]. The inability of the clonotypic antibody to recognize all CII-specific T cells in the Vβ12-tg mouse, however, prevented us from addressing the role of T cells in the later phases of arthritis development, by means of T-cell depletion studies. Depletion of B22a1^+ ^T cells after immunization with CII caused an even more rapid expansion (within days) of CII-specific B22a1^- ^T cells, and the depletion of B22a1^+ ^T cells was found to be less efficient in draining lymph nodes, compared with the spleen or blood (data not shown). Using combinatorial analysis of expression of the early activation marker CD40L and intracellular cytokines *ex vivo*, however, we could determine the frequency of CII-specific T cells as well as their cytokine profile at different time points after induction. Our data clearly showed that the frequency of CII-specific T cells peaked before onset of clinical disease. In line with this, the frequency of IFNγ-producing CII-specific T cells among CD4^+ ^T cells also peaked before onset of clinical arthritis and subsequently declined with progression of disease. In contrast, the frequency of CII-specific IL-17-producing T cells among CD4^+ ^T cells was less abundant in the early response, and instead increased in frequency to peak at the time of disease onset.

The role of IFNγ in CIA is complex and has been associated with both proinflammatory and regulatory functions [38-41], and more recent reports have challenged the concept that CIA is a Th1-mediated disease and have instead suggested the model to be more dependent on Th17 [42-45]. While determined *ex vivo *at the level of antigen-specific T cells, our findings on the relative abundance of IFNγ-responding and IL-17-responding T cells over time add support to the recent report by Lamacchia and colleagues in which investigations of the recall responses of bulk lymph node cells to CII in CIA indicated a shift from an initially dominant Th1 response to a mixed Th1/Th17 response [46]. Determining the role of CII-specific Th1 and Th17 in mediating effector functions and regulating the initiation or progression of CIA is challenging, however, as the lymphoid response varies over time and between lymphoid organs and the joints [47,48]. These factors, together with different techniques used for analyses of antigen-specific responses, probably help to explain some of the existing discrepancies with regard to which cell populations and cytokine responses predominate in lymphoid tissues and target organs during CIA [45-50]. Further analysis using the currently described animal model may help elucidate some of these matters. We have successfully used the B22a1 antibody for immunohistochemical identification of B22a1^+ ^T cells in lymph nodes and spleens of CII-primed Vβ12-tg mice (data not shown), and we are currently investigating the possibility to stain T cells residing in joints of arthritic animals.

## Conclusions

Because of the increased precursor frequency of CII-specific T cells in the Vβ12-tg mouse and the availability of an antibody recognizing the majority of these cells, the currently described animal model offers a unique possibility to further elucidate the role of CII-specific T cells as well as their different subsets during the development and regulation of CIA at different time points and in different tissues. The B22a1 antibody allows for highly sensitive analysis of joint-residing T cells specific for galactosylated CII by flow cytometry, due to its specificity and relatively strong affinity to its cognate TCR, compared with MHC class II tetramers.

## Abbreviations

APC: antigen-presenting cell; CDR3: complementarity determining region; CFA: complete Freund's adjuvant; CIA: collagen-induced arthritis; CII: collagen type II; DMEM: Dulbecco's modified Eagle's medium; ELISA: enzyme-linked immunosorbent assay; FCS: fetal calf serum; IFN: interferon; GALHYK264: CII(260-270) with a β-D-galactopyranosyl residue on L-hydroxylysine at position 264; GLCGALHYK264: CII(260-270) with an α-D-glucopyranosyl-(1 > 2)-β-D-galactopyranosyl residue on L-hydroxylysine at position 264; IL: interleukin; K264: CII(260-270) with a nonmodified lysine at position 264; PBS: phosphate-buffered saline; PCR: polymerase chain reaction; TCR: T-cell receptor; TG: transgenic; TH: T-helper cell.

## Competing interests

The authors declare that they have no competing interests.

## Authors' contributions

BS and HB contributed to the production of recombinant 22a1-7E TCR. RB contributed to the generation of T-cell hybridoma and sequencing of their CDR3 regions. PM, TB and BD contributed to CIA induction and evaluation. JB and PM contributed to generation and production of the monoclonal antibody B22a1. PM contributed to characterization of B22a1 specificity, flow cytometry analysis and T-cell stimulation analysis. PM and JB designed the study and drafted the manuscript, and all authors revised the manuscript. All authors read and approved the final manuscript.

## Supplementary Material

Additional file 1 **B22a1 antibody does not bind to T cells with other tested specificities than CII**. Wildtype mice were immunized with either pepsin or ovalbumin (OVA) emulsified in CFA. Seven days later, draining lymph node cells were restimulated *in vitro *with the immunogenic protein (third and fourth upper graphs from the left) as well as with purified protein derivate (tuberculin-purified protein derivative (PPD), mycobacterial antigens included in CFA; second upper panel from the left) or left unstimulated (upper left panel). After 6 hours of restimulation, cells were recovered and stained for expression of CD4, CD154 and B22a1. The A^q^-restricted pepsin-specific T-cell hybridoma HP1 was tested for staining with the B22a1 antibody (lower right panel), and staining of the 22a1-7E T-cell hybridoma clone was stained in parallel as positive control (lower left panel). Representative graphs for each restimulation condition is shown and numbers within the quadrants of the upper graphs shows the mean ± standard error of the mean of three individual mice per group immunized and restimulated *in vitro *with either pepsin or OVA and of all six mice restimulated *in vitro *with PPD or left unstimulated. Click here for file
